# Validation of a Sensorized Forearm Crutch for Quantifying Partial Weight-Bearing During Assisted Gait Using Optical Motion Capture and Instrumented Treadmill

**DOI:** 10.3390/s26134191

**Published:** 2026-07-02

**Authors:** Soufiane Mahraoui, Gerrit Bücken, Stefan Ecker, Syed Ibrahim Shakir, Arndt-Peter Schulz, Neki Muhametaj, Mauro Serpelloni

**Affiliations:** 1 Department of Information Engineering, University of Brescia, 25123 Brescia, Italy; soufiane.mahraoui@unibs.it (S.M.); n.muhametaj@studenti.unibs.it (N.M.); mauro.serpelloni@unibs.it (M.S.); 2 Fraunhofer Research Institution for Individualized Medical Engineering IMTE, 23562 Lübeck, Germany; gerrit.buecken@imte.fraunhofer.de (G.B.); stefan.ecker@imte.fraunhofer.de (S.E.); syed.ibrahim.shakir@imte.fraunhofer.de (S.I.S.); 3 Zentrum für Klinische Forschung (ZKF), BG Klinikum Hamburg, 21033 Hamburg, Germany

**Keywords:** crutch, instrumented crutches, gait cycle, ground reaction force, strain gauge, inertial measurement unit, assistive device, gait analysis

## Abstract

Human gait analysis is a key component of rehabilitation medicine, enabling objective assessment of patient recovery. In crutch-assisted locomotion, however, conventional forearm crutches operate as passive devices, providing no quantitative information on load distribution or patient adherence to partial weight-bearing (PWB) prescriptions. This work presents the design and dynamic validation of a sensorized forearm crutch system for biomechanical monitoring during assisted gait. The proposed device combines a force-sensing module based on a full Wheatstone bridge strain-gauge configuration with a 6-axis inertial measurement unit (IMU) to capture both axial load and crutch orientation. Sensor fusion was implemented through a complementary filter to estimate pitch and roll angles under dynamic conditions. The system was calibrated through static loading procedures and validated against reference instrumentation, including an optoelectronic motion capture system and an instrumented dual-belt treadmill with force platforms. Unlike previous studies relying on stationary force platforms that capture discrete steps and may alter natural gait, this validation approach enabled continuous, stride-by-stride force and orientation measurements without restricting foot placement. Experimental trials were conducted with unimpaired participants performing assisted gait using 2-point and 3-point patterns at two partial weight-bearing levels (20% and 40% body weight) and two walking speeds (0.80 m/s and 1.20 m/s). Dynamic validation showed good agreement with the treadmill reference, with force RMSE values of 9.33±1.70 N for the left crutch and 12.90±2.85 N for the right crutch, and with coefficients of determination of $R^{2}=0.9956$ and $R^{2}=0.9927$, respectively. Orientation RMSE values were 1.08±0.44° (roll, right), 2.06±0.56° (roll, left), 1.79±0.55° (pitch, right), and 1.66±0.37° (pitch, left). Beyond validation accuracy, the system enabled extraction of a set of quantitative biomechanical descriptors directly from crutch signals, axial load, cadence, crutch contact variability, load asymmetry, pitch asymmetry, and crutch stance/swing asymmetries, characterizing walking stability, bilateral coordination, and gait regularity during continuous assisted locomotion. These results demonstrate the feasibility of integrating force and inertial sensors into forearm crutches to enable quantitative monitoring of assisted gait, with potential applications in rehabilitation assessment and real-time feedback.

## 1. Introduction

Quantitative gait analysis is a fundamental component of modern rehabilitation medicine, providing objective information to support clinical assessment, treatment planning, and the monitoring of functional recovery. In many orthopedic and neurological conditions, however, patients do not walk independently but rely on assistive devices such as crutches, canes, or walkers. In these cases, the walking aid becomes an active part of the biomechanical system, contributing to load transfer, balance, stability, and compensatory motor strategies, and its characterization is therefore as important as lower-limb motion analysis [1]. Different assistive devices and gait strategies can affect walking speed, posture, support conditions, and stability, particularly in subjects with neurological impairments such as incomplete spinal cord injury [2], while crutch-assisted walking specifically modifies the distribution of mechanical loads across the body and increases the metabolic and mechanical demand on the upper limbs [3].

One of the main clinical challenges in crutch-assisted gait is the control of partial weight-bearing (PWB). Patients are instructed to load the affected limb with a prescribed fraction of body weight while transferring the remainder through the crutches [4]. Compliance with these prescriptions is difficult to enforce: excessive loading may compromise tissue healing or surgical fixation, whereas insufficient loading may impair recovery and contribute to muscle weakness [5]. In routine practice, PWB compliance is typically assessed through therapist observation or patient perception, approaches that are inherently subjective and unable to detect meaningful deviations from the target load. This limitation has motivated a growing line of research on instrumented walking aids capable of providing quantitative, objective information during assisted locomotion.

Early instrumented crutch systems integrated force sensors and IMUs to monitor load distribution and rehabilitation progress outside conventional laboratory settings [6,7]. These works demonstrated the feasibility of embedding sensing elements into standard forearm crutches, but they were primarily designed for monitoring purposes and were not subjected to rigorous quantitative validation against reference instrumentation. A more metrologically grounded approach was taken by Chamorro-Moriana et al. [8], who validated a compact force-sensing crutch against stationary force platforms. While this work established the reliability of the force measurement, the validation relied on discrete stance events captured by a fixed force plate, which inherently limits the number of analyzable steps per trial and is known to induce targeting behavior in which participants modify their step length to deliberately contact the plate surface, altering the natural walking pattern [9,10].

Subsequent systems expanded the sensing architecture to include inertial and wireless capabilities. Sesar et al. [11] developed an instrumented crutch tip capable of measuring axial force and estimating pitch angle, validating the orientation estimates against a Vicon motion capture system during walking. However, that study focused primarily on pitch estimation accuracy and did not provide simultaneous validation of both kinetic and kinematic signals during continuous gait. Sardini et al. [12,13] developed wireless instrumented crutches measuring axial and shear forces together with tilt angles, and they later extended the platform to support real-time biofeedback; validation was performed under controlled laboratory conditions but did not address continuous dynamic walking across multiple consecutive strides. Seylan et al. [14] proposed a low-cost instrumented crutch for estimating ground reaction force components from pressure and inclination data. While demonstrating feasibility for portable kinetic assessment, their estimation framework relied on a simplified quasi-static model that may not generalize to the impact dynamics and inertial perturbations characteristic of real gait.

Sensorized crutch tips have been proposed as minimally invasive alternatives for extracting functional indicators in persons with multiple sclerosis [15,16]. Spatial and temporal gait parameters derived from instrumented crutches have also been used for classification and characterization of different assisted-gait strategies [17,18], confirming that the walking aid can serve as an informative interface for objective user–device assessment. Design and feedback applications have further explored modifications of the crutch–ground interface [19], audio-guided PWB training, and smart crutches for exoskeleton-assisted rehabilitation [20]. More recent platforms integrate smart crutches with mobile health applications and haptic feedback, pointing toward patient-centered monitoring outside the laboratory [21,22]. In parallel, instrumented crutches have been integrated into wearable robotic systems for gait-phase detection, user-intention recognition, and exoskeleton control [23,24].

Despite the breadth of these developments, a critical limitation persists across most of the existing literature. The dynamic validation of instrumented crutches has not been addressed with the rigor required to establish their use as reliable measurement instruments. Most systems were validated under static, quasi-static, or tightly constrained walking conditions, or against stationary force platforms that capture only isolated, non-consecutive stance events. Crucially, none of the existing validation studies simultaneously evaluated both kinetic and kinematic performance, force and orientation, during continuous, stride-by-stride assisted walking. This gap is significant because dynamic walking introduces inertial perturbations, ground-impact transients, and time-varying crutch inclinations that are absent in bench calibrations and may substantially affect measurement accuracy in real use.

This work addresses this gap by presenting the design and dynamic validation of a sensorized forearm crutch system integrating a full Wheatstone bridge strain-gauge module and a 6-axis IMU. The primary contribution of this study is a comprehensive dynamic validation framework that simultaneously evaluates axial load and crutch orientation during continuous assisted gait against synchronized reference instrumentation. Unlike prior studies relying on stationary force platforms, validation was performed against a dual-belt instrumented treadmill combined with an optoelectronic motion capture system.

When applied to continuous assisted gait, stationary force plates present inherent constraints. Their restricted sensing area reduces the probability of obtaining valid foot contacts in a single pass, often requiring repeated non-consecutive runway trials that may not reflect steady-state gait [25]. Moreover, subjects may adopt targeting strategies to accurately strike the plate, potentially altering natural gait patterns [9,10]. Conventional force plate setups are therefore not well suited for continuous analysis of assisted walking, where multiple contacts from both lower limbs and assistive devices may occur in close temporal succession.

The dual-belt instrumented treadmill overcomes these limitations by enabling continuous, stride-by-stride bilateral force measurement at a precisely controlled and reproducible walking speed [26,27], without constraining foot or crutch placement. This provides a statistically robust reference that is more representative of continuous assisted gait than isolated force-plate steps, offering a practical ground truth for dynamic validation under controlled continuous gait conditions.

Beyond accuracy assessment, the validated system enables the extraction of a set of quantitative biomechanical descriptors, axial crutch load, cadence, crutch contact variability, load asymmetry, pitch asymmetry, and crutch stance/swing asymmetries, which are commonly used to characterize gait dynamics in terms of temporal variability, stability, and inter-limb coordination [28,29,30]. These features provide a compact kinematic and kinetic representation of continuous assisted gait, supporting the objective evaluation of the sensorized forearm crutch and enabling partial weight-bearing quantification during assisted walking.

## 2. Materials and Methods

### 2.1. Instrumented Crutches

**Figure 1:**
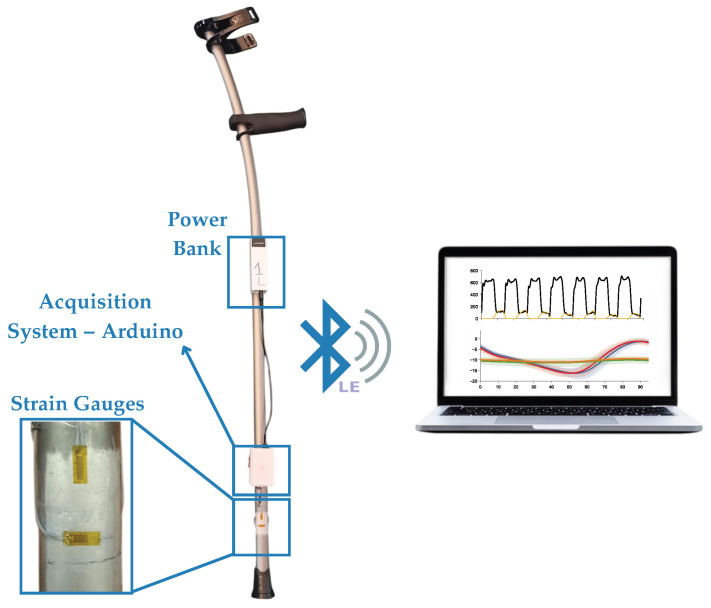
Schematic representation of the instrumented crutch system.

A pair of commercial forearm crutches was instrumented to acquire the main biomechanical quantities required for the dynamic validation of assisted gait, namely the axial load transmitted through each crutch and its spatial orientation during walking. A schematic overview of the system architecture is shown in Figure 1.

Each crutch was equipped with a full Wheatstone bridge composed of four strain gauges bonded to the lower portion of the shaft. This configuration was used to estimate the axial force transmitted through the crutch during ground contact, while reducing the influence of temperature variations and parasitic bending effects. The bridge output was amplified by a conditioning circuit and acquired by an Arduino Nano 33 BLE Rev2 (Arduino, Somerville, MA, USA) board. The same board also included a Bosch BMI270 (Bosch Sensortec GmbH, Reutlingen, Germany) 6-axis inertial measurement unit, composed of a triaxial accelerometer and a triaxial gyroscope.

Force and inertial data were sampled locally at 50 Hz. Data from the two crutches were transmitted wirelessly to a host computer using Bluetooth Low Energy (BLE) and recorded through a Python-based acquisition software (version 3.13, Python Software Foundation, Wilmington, DE, USA). Each transmitted packet included a local timestamp generated by the microcontroller, allowing the temporal sequence of the signals to be reconstructed during post-processing.

Crutch orientation was estimated in terms of pitch and roll angles. Under dynamic conditions, orientation estimation was performed using a complementary filter, based on the method described by Pedley [31]:

(1) $$\theta \left(t\right)=\alpha \;\theta_{\mathrm{gyro}}\left(t\right)+\left(1-\alpha \right)\;\theta_{\mathrm{acc}}\left(t\right)$$

(2) $$\phi \left(t\right)=\alpha \;\phi_{\mathrm{gyro}}\left(t\right)+\left(1-\alpha \right)\;\phi_{\mathrm{acc}}\left(t\right)$$

where θ(t) and ϕ(t) are the estimated pitch and roll angles, and α=0.97, consistent with values reported for MEMS-based inertial sensor fusion [31,32]. This value reflects a trade-off between the gyroscope’s short-term accuracy and its long-term drift, and the accelerometer’s long-term stability but sensitivity to motion-induced accelerations during dynamic crutch use. Weighting the gyroscope at 97% preserves responsiveness during fast movements, while the remaining 3% from the accelerometer is enough to correct drift over time.

### 2.2. Python-Based GUI

**Figure 2:**
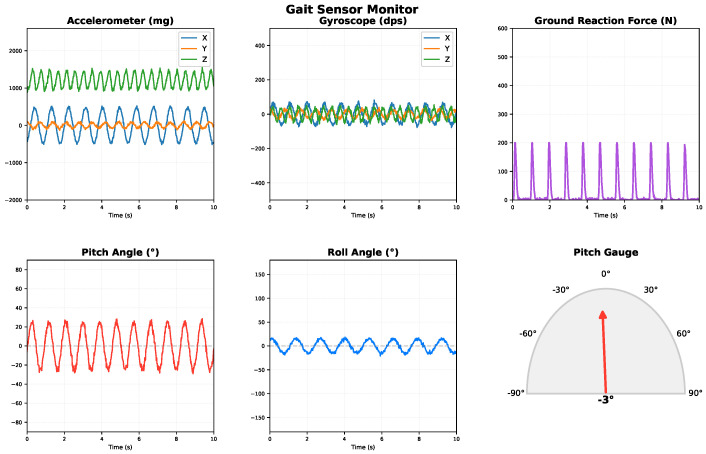
Python-based GUI for real-time acquisition and visualization of the instrumented crutches signals.

A Python-based graphical user interface (GUI) was developed to enable the simultaneous connection of two Arduino-based acquisition units, allowing both instrumented crutches to transmit data to the PC concurrently. The GUI controls the acquisition procedure via Bluetooth communication and allows the operator to scan for and connect to the devices, start and stop the recording, and export the acquired data in CSV format for subsequent offline processing. The software architecture and implementation details adopt the framework described in our earlier work [33]. As shown in Figure 2, the interface provides real-time visualization of the acquired signals from both instrumented crutches.

### 2.3. Strain Gauges Static Calibration

**Figure 3:**
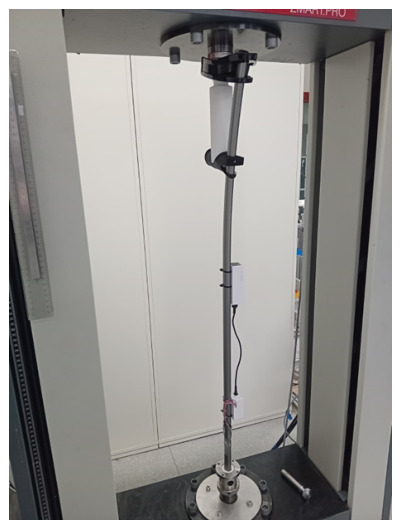
Static calibration setup.

Before the dynamic validation experiments, a static calibration procedure was per-formed to convert the Wheatstone bridge output voltage into axial force. The calibration was carried out using a calibrated dynamometer equipped with a 50 kN load cell, as shown in Figure 3. During the test, each instrumented crutch was mounted vertically and aligned with the loading axis of the dynamometer in order to reproduce an axial compression condition comparable to the load transmitted through the crutch during ground contact. The crutch was subjected to a quasi-static axial load in the range of 0–600 N, applied at a test speed of 1 mm/min. The reference force measured by the dynamometer load cell was acquired at 100 Hz and used as the calibration reference for the strain gauge measurement system. Seven calibration trials were performed for the left crutch, while six trials were performed for the right crutch. The relationship between the applied axial force and the Wheatstone bridge output voltage was modeled using linear regression. Separate calibration models were obtained for the left and right crutches, since small differences in strain-gauge bonding, positioning, or mechanical response of the shaft may produce different sensitivities between the two devices. The obtained calibration coefficients were then used in the subsequent dynamic validation analyses to estimate the axial force transmitted through each instrumented crutch.

### 2.4. Validation System: Vicon Optical System and Motek Treadmill

**Figure 4:**
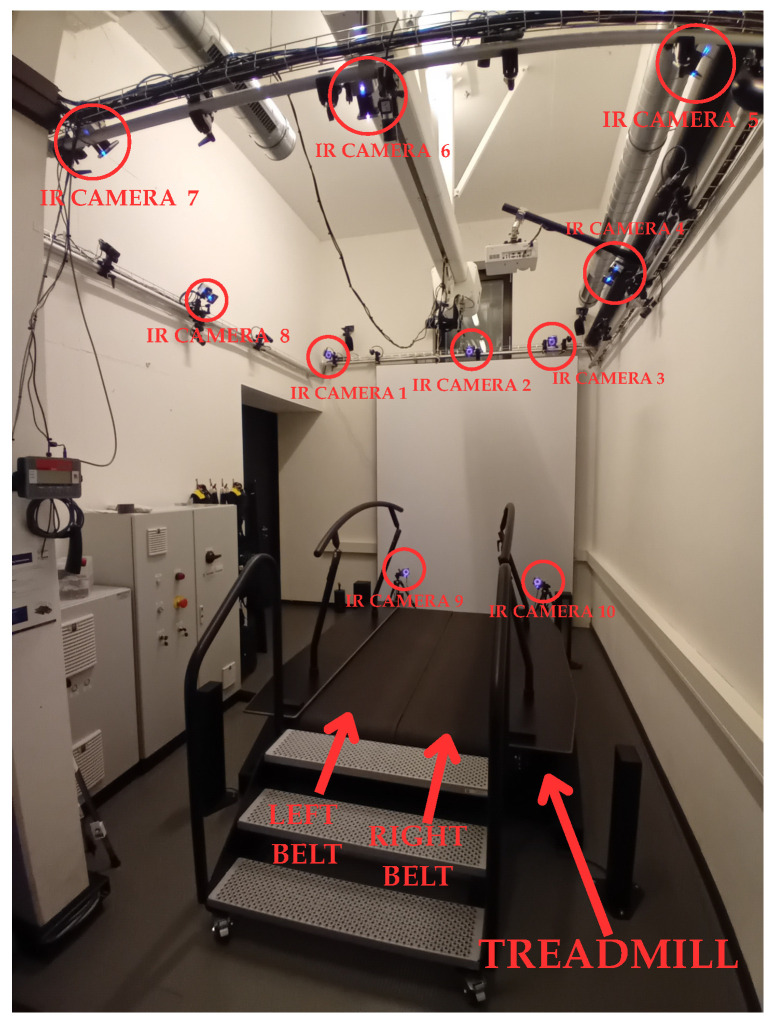
Laboratory setup with 10 infrared Vicon cameras surrounding the Motek dual-belt instrumented treadmill.

The validation of the instrumented crutches was conducted in two stages. First, a static validation was performed to assess the accuracy of the IMU orientation estimates under controlled, stationary conditions. Subsequently, a dynamic validation was carried out during assisted walking trials to evaluate the system performance under realistic operating conditions. Both stages relied on a Vicon (Vicon Motion Systems, Oxford, UK) optical motion capture system integrated with a Motek dual-belt instrumented treadmill, as shown in Figure 4. The treadmill was positioned at the center of the laboratory and surrounded by ten infrared Vicon cameras, which acquired the three-dimensional motion of the crutches at a sampling frequency of 100 Hz.

The Motek treadmill consisted of two independent belts, each integrating a 6-DOF force platform. Ground reaction forces were acquired at 1000 Hz and used as the reference for the validation of the force measured by the instrumented crutches. The optical and treadmill signals were automatically synchronized within the Vicon–Motek acquisition system.

**Figure 5:**
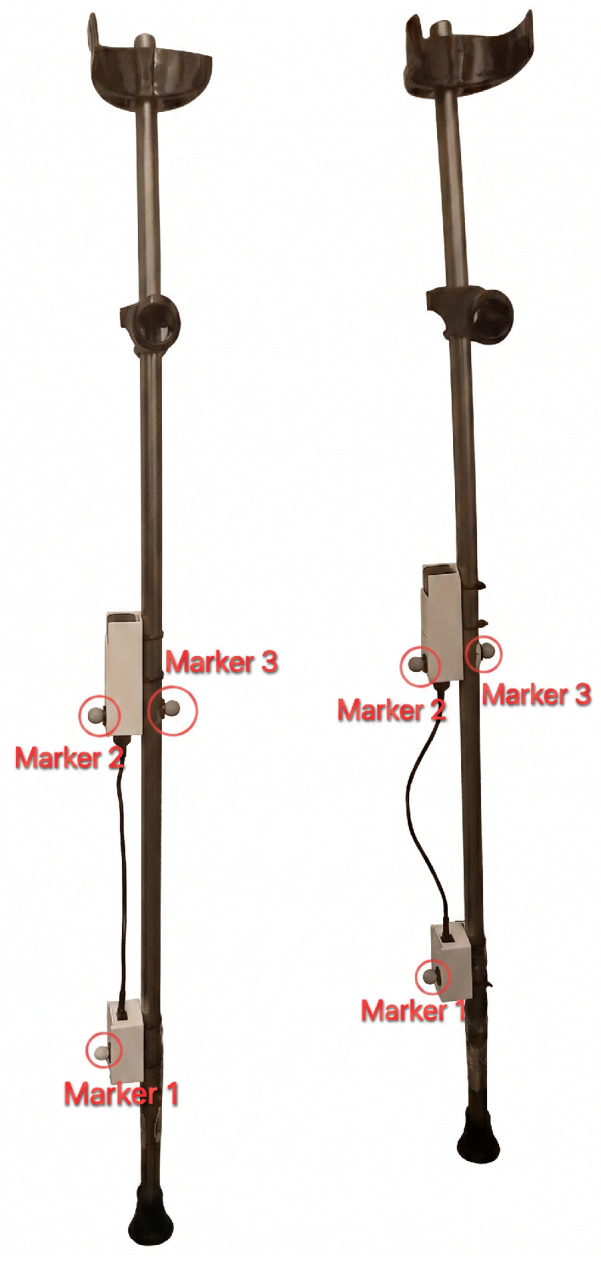
Instrumented crutch with three passive reflective markers for optical tracking.

Three passive reflective markers were mounted on each instrumented crutch to define a rigid body for optical tracking (Figure 5). One marker was positioned above the Arduino enclosure and used as the origin of the local crutch reference frame. The remaining markers were placed along the crutch shaft to obtain a non-collinear configuration, enabling the reconstruction of crutch orientation during walking.

#### 2.4.1. Static Validation

The static validation was carried out using the Vicon system described above as the reference for crutch orientation. Four static exercises were performed using one instrumented crutch. The exercises were designed to evaluate pitch and roll angle estimation separately, by varying only one angular component at a time while keeping the other approximately constant.

Two exercises were performed for the roll angle. In the first exercise, the crutch was statically positioned at different intermediate angular values while varying the roll angle from −90° to +90°. In the second exercise, the same procedure was repeated over a reduced angular range, from −45° to +45°. The same procedure was then repeated for the pitch angle.

#### 2.4.2. Dynamic Validation

The dynamic validation was performed during assisted walking trials on the instrumented treadmill. Each instrumented crutch logged force and orientation data at 50 Hz. The Vicon optical system acquired crutch orientation at 100 Hz, while the Motek treadmill recorded ground reaction forces at 1000 Hz. To allow for point-to-point comparison, both reference signals were resampled to 50 Hz after low-pass filtering to prevent aliasing, thereby matching the sampling rate of the crutches.

Temporal alignment between the crutch and reference signals was performed independently for each trial using normalized cross-correlation between the strain-gauge force signal and the vertical ground reaction force measured by the treadmill. The lag maximizing the cross-correlation was identified and applied to shift the reference signals, ensuring precise sample-level synchronization across all measurement systems. The same temporal offset was applied to the Vicon orientation data, resulting in a common synchronized time base for all kinetic and kinematic metrics.

### 2.5. Experimental Protocol

**Figure 6:**
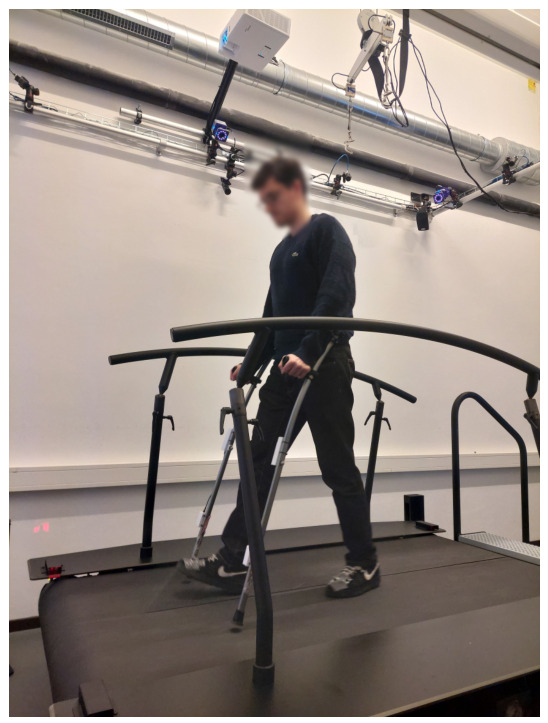
Representative example of a participant performing an assisted-gait trial on the instrumented dual-belt treadmill, surrounded by the Vicon infrared camera array.

The proposed tasks were selected to reproduce representative crutch-assisted walking conditions, including non-weight-bearing and partial-weight-bearing strategies commonly adopted during rehabilitation. A preliminary walking trial without crutches was included to obtain the reference body weight, corresponding to 100% BW, which was then used to define the prescribed partial-weight-bearing targets. Walking speed was imposed by the instrumented treadmill and set to 1.20 m/s for the unaided walking condition and to 0.80 m/s for the crutch-assisted walking conditions, using the study by Kingston et al. [34] as a methodological reference. Before each experimental task, the participants completed a familiarization phase with the required walking condition. Its duration was not fixed and was left to the participant’s discretion, allowing each participant to become comfortable with the task before data acquisition. Figure 6 shows a representative example of a participant performing an assisted-gait trial during data acquisition.

#### 2.5.1. Strain Gauges Validation

The dynamic validation of the strain-gauge subsystem was performed against the vertical ground reaction force measured by the instrumented treadmill. A swing-through non-weight-bearing gait was selected to isolate the load transmitted through the crutches and to minimize the contribution of the lower limbs to the treadmill force signal.

Two experimental conditions were tested, swing-through non-weight-bearing gait with the left leg lifted and swing-through non-weight-bearing gait with the right leg lifted. For each condition, five trials were performed at a walking speed of 0.80 m/s, with a duration of approximately 20 s per trial. This procedure enabled the dynamic response of the Wheatstone bridge force estimation to be assessed under controlled crutch-loading conditions. The experimental tasks are summarized in Table 1.

#### 2.5.2. IMU Dynamic Validation Setup

The experimental tasks reported in Table 2 were designed to extract the biomechanical metrics analyzed in Section 3.5 and to perform the dynamic validation of the IMU-based orientation estimation. The pitch and roll angles estimated from the instrumented crutches were compared with the corresponding crutch orientations reconstructed from the Vicon marker trajectories.

The first task was an unaided walk, performed without crutches at 1.20 m/s. This condition was used to determine the reference body weight, corresponding to 100% BW, from the vertical ground reaction force measured by the instrumented treadmill. For each participant, 100% BW was obtained by considering the first peak of the vertical ground reaction force to be equal to 110% BW, in accordance with the values reported by Keller et al. [35]. This reference value was then used to express the strain-gauge force measurements as a percentage of body weight (%BW).

The second and third tasks consisted of 3-point gait at 0.80 m/s, with prescribed crutch loads of 20% BW and 40% BW, respectively. In this gait pattern, both crutches and the unloaded or partially loaded lower limb are advanced first, followed by the contralateral limb. The prescribed load levels were selected to simulate clinically relevant partial weight-bearing instructions and to assess the participants’ ability to comply with the assigned crutch-loading targets.

The prescribed PWB targets (20% and 40% BW) refer to the total load to be transmitted through both crutches combined, rather than to each crutch individually.

The fourth and fifth tasks consisted of 2-point gait, performed at 0.80 m/s and 1.20 m/s. In this gait pattern, each crutch is advanced simultaneously with the contralateral lower limb, producing a more alternating and continuous assisted-walking strategy. The two walking speeds were used to evaluate the effect of increased gait velocity on the extracted kinetic, kinematic, and temporal parameters.

All tasks were performed by 5 unimpaired participants. Each trial lasted approximately 10 s and was performed once by each participant. The experimental tasks are summarized in Table 2.

### 2.6. Gait Metrics and Load Analysis

The signals acquired from the instrumented crutches were processed to extract kinetic, kinematic, and temporal parameters characterizing assisted gait. Crutch–ground contact events were identified from the strain-gauge force signal using a threshold of 5 N and a minimum contact duration of 500 ms to reject spurious events. Each cycle was segmented into a stance phase, representing the interval of ground contact, and a swing phase, representing the interval while the crutch was airborne. All force measurements were normalised to body weight (%BW) using the reference value obtained from the unaided walking trial (Section 2.5).

The selected metrics capture complementary aspects of crutch-assisted locomotion. Crutch load quantifies the mechanical demand placed on the upper limbs and reflects the level of body-weight support provided by the device; adequate monitoring of this parameter is clinically relevant since compliance with partial weight-bearing prescriptions is frequently not achieved without objective feedback [6,7]. Pitch and roll angles describe crutch orientation during ground contact, providing information on crutch placement strategies and supporting the estimation of fall risk and detection of gait irregularities [11,13]. Cadence and crutch contact variability characterize the temporal organization and consistency of crutch usage; increased variability in crutch inter-contact intervals in particular has been identified as a sensitive indicator of reduced stability and impaired motor control [28,36].

To assess bilateral coordination, asymmetry indices were computed from paired left and right crutch events. In 3-point gait, pairs were defined by temporal overlap, while in 2-point gait consecutive alternating contacts were used. Load and pitch asymmetry reflect unequal weight distribution and orientation between the crutches, respectively [13,17]. For 2-point gait, temporal symmetry was further evaluated through crutch stance and swing asymmetry, quantifying the bilateral coordination of crutch–ground contact phases; marked differences between sides have been associated with less stable or irregular gait patterns in crutch-assisted locomotion [18,36]. All metrics reported here are derived exclusively from crutch contact signals and characterize crutch movement patterns, not lower-limb gait variables. The extracted metrics are summarized in Table 3.

## 3. Results

### 3.1. Strain Gauges Calibration

The axial load calibration for both crutches demonstrated high linearity across the full measurement range, with individual regressions yielding $R^{2}=0.999$. The slopes were 1.074 mV/N (95% CI: [1.038;1.111] mV/N) for the left crutch and 0.862 mV/N (95% CI: [0.837;0.886] mV/N) for the right crutch. A Welch two-sample *t*-test confirmed that the difference in sensitivity between the two devices was statistically significant (t=11.96, p<0.001), and pooling the data reduced agreement to $R^{2}=0.987$, confirming that device-specific calibration coefficients are required for accurate force estimation (Table 4).

### 3.2. IMU Static Validation

Static validation against the Vicon optical motion capture system showed that estimation accuracy is sensitive to the angular range. Within the operationally relevant range of ±45°, the MAE was 1.61° (SD: 1.53°) for roll and 2.47° (SD: 3.13°) for pitch. Errors increased substantially over the full ±90° range, with larger deviations near extreme angular positions. Detailed results are reported in Table 5.

### 3.3. Strain Gauges Dynamic Validation

**Figure 7:**
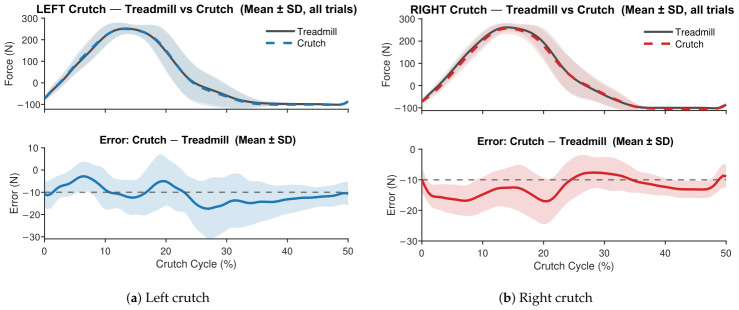
Normalized crutch cycle force profiles. Shaded areas represent the standard deviation across detected cycles.

The force profiles were analyzed over normalized crutch cycles, defined as the interval between two consecutive ground contacts of the same crutch. Each cycle was normalized from 0% to 100% to allow comparison between the instrumented crutch signal and the treadmill reference, as shown in Figure 7.

**Figure 8:**
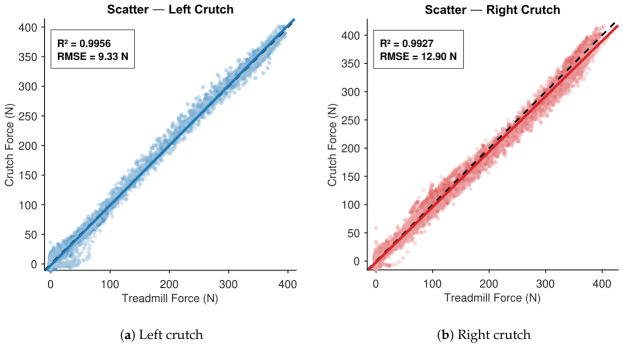
Scatter plots between the instrumented crutch forces and the treadmill reference.

Force peaks occurred in the same phase of the crutch cycle for both the strain-gauge measurements and the treadmill reference. The scatter plots in Figure 8 show the agreement between the two systems over the investigated force range.

The coefficient of determination was $R^{2}=0.9956$ for the left crutch and $R^{2}=0.9927$ for the right crutch. The quantitative results are summarized in Table 6. The left crutch showed an MBE of −0.70±3.17 N and an RMSE of 9.33±1.70 N (LoA: [−26.54;16.88] N), while the right crutch showed an MBE of −4.83±6.28 N and an RMSE of 12.90±2.85 N (LoA: [−19.88;18.47] N). Considering a maximum force range of approximately 400 N, the RMSE corresponded to 2.3% and 3.2% of the measured force range for the left and right crutch, respectively.

### 3.4. IMU Dynamic Validation

**Figure 9:**
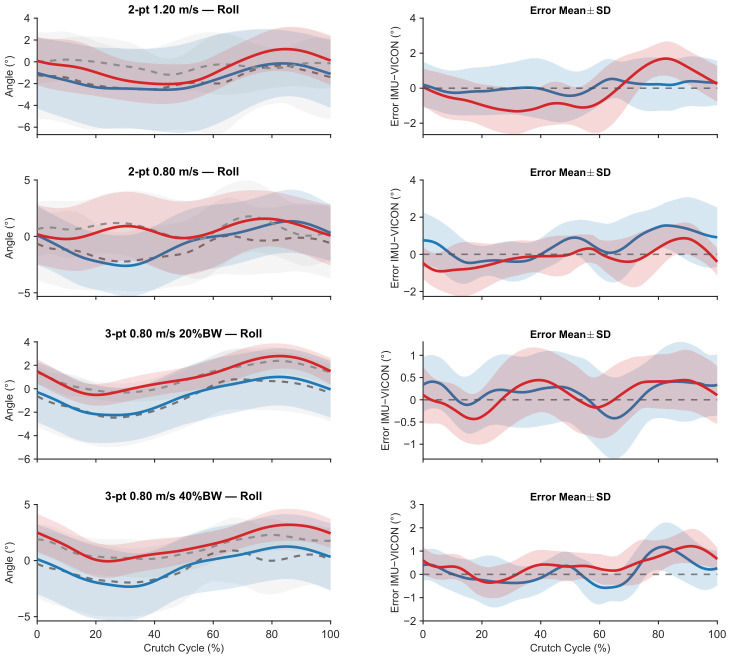
Roll angle profiles over the normalized crutch cycle (0–100%): mean ± SD for the IMU (solid line) and Vicon reference (dashed line), comparing left (blue) and right (red) crutches across all gait patterns.

**Figure 10:**
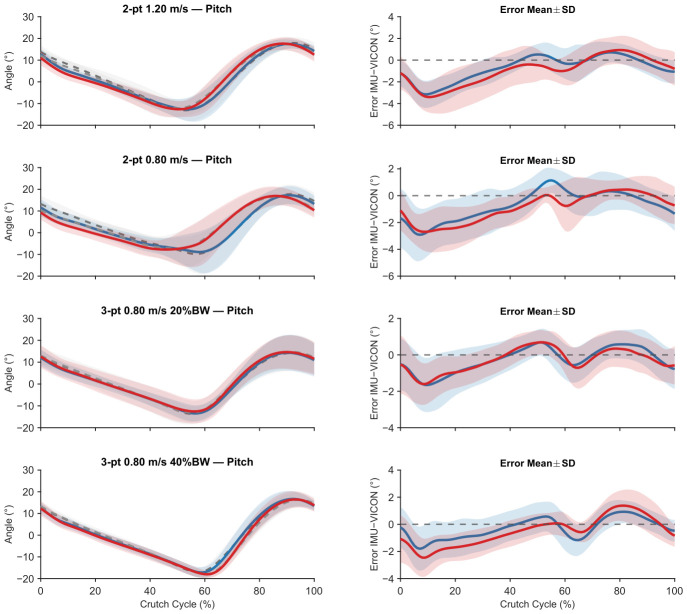
Pitch angle profiles over the normalized crutch cycle (0–100%): mean ± SD for the IMU (solid line) and Vicon reference (dashed line), comparing left (blue) and right (red) crutches across all gait patterns.

The pitch and roll profiles over normalized crutch cycles are shown in Figure 9 and Figure 10 for all gait patterns.

The quantitative results are summarized in Table 7. For roll, the MBE was 0.07±0.58° (right) and 0.15±0.39° (left), with RMSE of 1.08±0.44° and 2.06±0.56°, respectively, and LoA of [−1.94;2.09]° and [−1.82;2.12]°. For pitch, the MBE was −0.73±0.61° (right) and −0.58±0.58° (left), with RMSE of 1.79±0.55° and 1.66±0.37°, respectively, and LoA of [−3.84;2.44]° and [−3.30;2.37]°.

### 3.5. Biomechanical Metrics

The biomechanical metrics extracted during the experimental tasks are defined in Table 3 and their values reported in Table 8.

**Figure 11:**
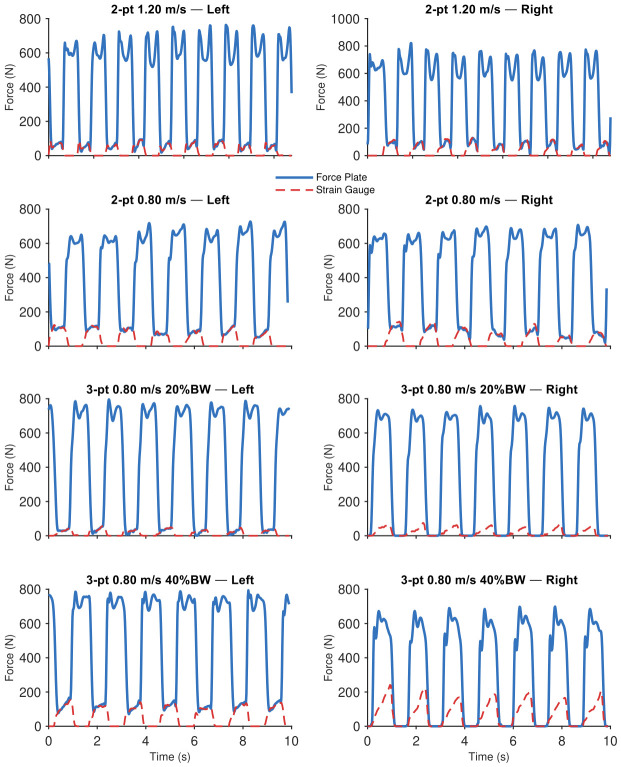
Comparison between treadmill force plates and crutch forces during the different assisted-gait tasks.

In the 3-point gait trials, the crutch loads increased from 9.42±4.46% BW and 11.57±4.98% BW at 20% BW to 21.15±8.81% BW and 27.92±12.30% BW at 40% BW for the left and right crutch respectively, with the right crutch consistently loaded more than the left. As an illustration of the type of compliance information obtainable from the combined crutch signals, the summed left and right loads were 20.99% BW at the 20% BW target and 49.07% BW at the 40% BW target, exemplifying how such a metric could be used in future applications to flag deviations from prescribed PWB instructions. Load asymmetry was largest in 3-point gait at 40% BW (7.21±4.02% BW) and lower in 2-point gait (≤4.12% BW). Cadence ranged from 75.86±8.41 steps/min in 2-point gait at 0.80 m/s to 94.09±6.34 steps/min at 1.20 m/s, while crutch contact variability and crutch stance/swing asymmetries showed high standard deviations across participants. Pitch values remained consistent across all conditions, and roll angles stayed within a limited range throughout. Figure 11 shows the raw force signals from the treadmill force plates and the strain-gauge estimates across all experimental tasks, allowing visual identification of the four gait conditions.

## 4. Discussion

The static and dynamic validation results demonstrate that the proposed instrumented crutches provide reliable measurements of both axial force and orientation under conditions representative of assisted gait.

The calibration procedure revealed linear and repeatable responses for both devices, supporting the robustness of the sensing architecture. However, the two crutches exhibited significantly different sensitivities. This finding indicates that identical force loads produce different electrical outputs in the two devices, making a common calibration equation unsuitable. The reduced agreement observed when pooling calibration data from both crutches further confirms that independent calibration coefficients are mandatory to prevent systematic errors in force estimation [13]. Notably, these sensitivity differences did not affect the estimation of bilateral loading asymmetry, as each device was calibrated independently. Force metrics were derived from values already converted into physical units (N) using device-specific equations, thereby eliminating any influence of sensor-to-sensor variability on symmetry calculations.

Under dynamic walking conditions, force measurements showed high agreement with the instrumented treadmill reference. The low RMSE values, high coefficients of determination ($R^{2}$), and consistent temporal alignment of points of interest indicate that the sensing subsystem accurately captures the kinetics of crutch loading. This precision is essential for the objective quantification of weight-bearing and for the reliable identification of loading events within the gait cycle. Overall, the measurement performance is comparable to values reported for previous instrumented crutch systems [13,37].

The swing-through non-weight-bearing condition was deliberately selected for strain-gauge validation because it spans the full range of axial loads transmitted through the crutch, from near-zero during the swing phase to values approaching full body-weight transfer during stance. As shown in the scatter plots (Figure 8), the agreement between crutch and treadmill forces remained high ($R^{2}>0.99$) across this entire range, including the lower force magnitudes (approximately 20–40% BW) corresponding to the partial weight-bearing levels used in the 2-point and 3-point gait trials. The validated accuracy can therefore be considered representative of the force ranges encountered across all gait conditions analyzed in this study.

Regarding orientation estimation, static validation confirmed that the IMU achieves its highest accuracy within the angular range typically encountered during assisted walking. Dynamic validation showed pitch accuracy comparable to literature values [11]. The high relative error observed in the roll axis was primarily a consequence of the small range of motion recorded during these specific trials (<5°), where even minimal absolute noise represents a large percentage of the total movement. Nevertheless, the roll signal remains fundamental for safety-related applications, as the ability to detect sudden and large changes in lateral inclination is essential for identifying anomalous events such as a crutch slipping or a potential fall. The negligible mean bias across both axes further confirms that these orientation estimates are stable and suitable for both quantitative gait characterization and the detection of atypical loading or placement patterns.

The dual-belt treadmill provided a rigorous reference for validating crutch-assisted gait. Furthermore, the synchronization of kinetic data with the Vicon system allowed for a comprehensive multimodal validation within a unified experimental framework.

The biomechanical metrics extracted from the validated force and orientation signals provide an initial demonstration of the range of information that can be obtained from the proposed instrumented crutches during continuous assisted gait. The measured parameters were able to discriminate between the investigated gait conditions and reflected the expected effects of different loading prescriptions and walking strategies.

The force measurements showed a clear increase in crutch loading between the two 3-point gait conditions. The combined load supported by the crutches increased from 20.99% BW during the 20% BW task to 49.07% BW during the 40% BW task, demonstrating the capability of the system to objectively quantify partial weight-bearing behavior. While the participants closely matched the prescribed target in the 20% BW condition, a tendency toward higher-than-prescribed loading was observed in the 40% BW condition. These findings illustrate the potential of the proposed system to identify deviations from prescribed weight-bearing instructions and to provide objective information on load distribution during assisted locomotion.

Temporal parameters were also sensitive to changes in gait conditions. Cadence increased from 75.86 steps/min during 2-point gait at 0.80 m/s to 94.09 steps/min at 1.20 m/s, reflecting the expected adaptation to increased walking speed. Crutch contact variability and crutch stance/swing asymmetry exhibited relatively large standard deviations across participants, indicating a substantial inter-participant variability in crutch-handling strategies despite the use of standardized gait patterns.

The asymmetry metrics further highlighted differences between gait conditions. Load asymmetry was greatest during the 3-point gait at 40% BW, suggesting that higher upper-limb loading may amplify side-to-side differences in support distribution. In contrast, lower asymmetry values were observed during the 2-point gait conditions, indicating a more balanced bilateral use of the crutches.

The orientation measurements revealed relatively consistent pitch patterns across all tasks. Peak pitch values remained within a similar range regardless of gait strategy or walking speed, suggesting that participants adopted comparable crutch placement mechanics throughout the experiments. Roll excursions remained limited, generally within a few degrees from the neutral position, which is consistent with the constrained lateral motion expected during stable crutch-assisted walking.

Although the present study was not designed to investigate clinical outcomes, these results demonstrate that the validated sensing platform can provide a comprehensive set of temporal, kinetic, and kinematic descriptors from instrumented crutches alone. Such information could support future studies aimed at quantifying weight-bearing compliance, gait symmetry, walking stability, and adaptations to different assisted-gait strategies.

In future clinical use, a sensorized forearm crutch could become a practical tool for monitoring and guiding partial weight-bearing during gait rehabilitation, particularly after orthopedic surgery or lower-limb injury. By providing objective load measurements, it could help clinicians ensure that patients adhere to prescribed unloading targets, reducing the risk of overloading, underloading, and poor gait symmetry. Its primary application would be in physiotherapy clinics and rehabilitation laboratories, where therapists could individualize targets, track progress objectively, and adjust feedback to each patient’s needs. With further validation, the system could also support home-based or community walking, making recovery more continuous and better connected to real-world mobility.

Some limitations of the present study should be acknowledged. The validation was conducted on a small sample of five unimpaired participants, which limits generalizability to clinical populations with orthopedic or neurological impairments. Additionally, testing was performed exclusively on a treadmill, which does not fully reproduce overground or real-world walking conditions. Further validation in larger and more representative clinical samples, as well as in overground and community-based settings, is therefore warranted.

## 5. Conclusions

This study presented and validated a pair of instrumented forearm crutches for the continuous and synchronous measurement of axial load and crutch orientation during assisted gait. The integration of strain-gauge-based force sensing and inertial measurement units within the crutch frame enables the acquisition of kinetic and kinematic parameters in a single, wearable platform.

Device-specific calibration was found to be mandatory due to significant inter-device sensitivity differences. Dynamic validation against an instrumented dual-belt treadmill and an optical motion capture system confirmed high accuracy for both force ($R^{2}>0.99$) and orientation estimation, with performance consistent with previously reported instrumented crutch systems.

Beyond metrological performance, the system demonstrated the ability to discriminate between gait conditions and to detect deviations from prescribed partial weight-bearing targets, providing a comprehensive set of temporal, kinetic, and kinematic descriptors from a single platform. The substantial inter-participant variability observed even in unimpaired participants underlines the importance of objective, continuous monitoring in assisted gait rehabilitation.

Future work will focus on validating the system in clinical populations, including patients after orthopedic surgery or lower-limb injury, to assess its performance across a wider range of pathological gait patterns and loading asymmetries. A larger and more diverse sample of participants will be recruited to improve the generalizability of the findings. Validation under overground and real-world conditions will also be pursued to overcome the ecological constraints of treadmill-based testing. The implementation of real-time feedback mechanisms will be investigated, with the long-term goal of supporting home-based recovery and connecting rehabilitation to real-world mobility.

## Figures and Tables

**Table 1:** Strain-gauge dynamic validation protocol.

Gait Condition	Speed	Trials	Duration
Swing-through gait, left foot lifted	0.80 m/s	5	∼20 s
Swing-through gait, right foot lifted	0.80 m/s	5	∼20 s

**Table 2:** IMU validation and biomechanical metrics protocol.

Gait Condition	Participants	Speed	Prescribed Load	Duration
Unaided	5 unimpaired participants	1.20 m/s	—	∼10 s
3-point gait	5 unimpaired participants	0.80 m/s	20% BW on crutches	∼10 s
3-point gait	5 unimpaired participants	0.80 m/s	40% BW on crutches	∼10 s
2-point gait	5 unimpaired participants	0.80 m/s	—	∼10 s
2-point gait	5 unimpaired participants	1.20 m/s	—	∼10 s

**Table 3:** Biomechanical descriptors used for the characterization of crutch-assisted gait.

Metric	Unit	Description	Clinical Relevance
Crutch Load	%BW	Mean peak force during crutch stance phase.	Assessment of weight-bearing compliance [37].
Pitch/Roll	°	Mean per-cycle angular peaks (max/min).	Evaluation of crutch placement stability and fall risk [13].
Cadence	steps/min	Total crutch contacts detected divided by trial duration.	Global descriptor of temporal crutch usage performance.
Crutch Contact Variability	ms	Standard deviation of inter-contact intervals of the crutch.	Marker of irregularity in crutch temporal pattern [28].
Load Asymmetry	%BW	Mean bilateral difference in peak crutch load.	Identification of compensatory movement strategies [17].
Pitch Asymmetry	°	Mean bilateral difference in crutch pitch peaks.	Side-to-side inconsistency in crutch handling [13].
Crutch Stance/Swing Asymmetry *	ms	Mean bilateral difference in crutch stance/swing phase durations.	Evaluation of temporal coordination in crutch usage [18].

* Defined for 2-point gait only.

**Table 4:** Calibration parameters for individual and combined regressions.

Device	Slope [mV/N]	95% CI [mV/N]	$R^{2}$
Left Crutch	1.074	[1.038;1.111]	0.999
Right Crutch	0.862	[0.837;0.886]	0.999
Combined Dataset	0.976	[0.907;1.046]	0.987

**Table 5:** Static IMU validation errors for roll and pitch angles.

Condition	MAE (°)	SD (°)
Roll–Reduced Range (±45°)	1.61	1.53
Roll–Full Range (±90°)	4.90	6.09
Pitch–Reduced Range (±45°)	2.47	3.13
Pitch–Full Range (±90°)	5.25	7.32

**Table 6:** Dynamic validation results of the strain-gauge force measurement system against the instrumented treadmill reference.

Device	MBE (N) ± SD	RMSE (N) ± SD	LoA (N)	$R^{2}$
Left Crutch	−0.70±3.17	9.33±1.70	[−26.54;16.88]	0.9956
Right Crutch	−4.83±6.28	12.90±2.85	[−19.88;18.47]	0.9927

**Table 7:** Dynamic validation results for angle estimation against the Vicon optical reference system.

Device	Angle	MBE (°) ± SD	RMSE (°) ± SD	LoA (°)
Left crutch	Roll	0.15±0.39	2.06±0.56	[−1.82;2.12]
	Pitch	−0.58±0.58	1.66±0.37	[−3.30;2.37]
Right crutch	Roll	0.07±0.58	1.08±0.44	[−1.94;2.09]
	Pitch	−0.73±0.61	1.79±0.55	[−3.84;2.44]

**Table 8:** Biomechanical metrics extracted during the different assisted-gait tasks.

Parameter	3-pt 20% BW	3-pt 40% BW	2-pt 0.80 m/s	2-pt 1.20 m/s
Force Left (%BW)	9.42±4.46	21.15±8.81	14.37±3.96	13.64±6.77
Force Right (%BW)	11.57±4.98	27.92±12.30	16.23±3.19	15.54±5.02
Cadence (steps/min)	81.84±3.06	81.85±3.66	75.86±8.41	94.09±6.34
Crutch Contact Variability (ms)	45.51±27.98	37.48±16.05	97.18±85.98	55.41±25.56
Load Asymmetry (%BW)	2.78±1.27	7.21±4.02	4.12±1.42	3.80±0.46
Pitch Asym.–Pos. Peak (°)	4.80±1.87	2.21±0.64	3.10±1.29	2.42±1.90
Pitch Asym.–Neg. Peak (°)	3.09±0.74	1.86±0.72	3.33±3.14	2.86±1.02
Pitch Max–Left (°)	16.59±3.11	17.11±3.68	18.25±3.35	18.22±1.77
Pitch Max–Right (°)	17.55±3.18	16.89±3.79	17.35±4.49	18.20±2.68
Pitch Min–Left (°)	−14.76±1.38	−17.76±0.58	−12.63±3.64	−14.53±2.02
Pitch Min–Right (°)	−14.95±3.94	−18.52±1.55	−11.85±3.61	−14.75±1.88
Roll Max–Left (°)	1.33±2.36	1.41±3.17	1.84±2.83	0.46±3.18
Roll Max–Right (°)	3.14±0.50	3.55±1.54	1.81±2.59	1.32±2.04
Roll Min–Left (°)	−2.41±2.46	−2.52±3.09	−2.55±2.69	−2.89±3.96
Roll Min–Right (°)	−0.73±0.54	−0.20±0.73	−1.33±2.71	−2.36±1.72
Crutch Swing Asymmetry (ms)	—	—	103.17±92.58	58.20±20.73
Crutch Stance Asymmetry (ms)	—	—	89.35±82.26	32.67±13.03

## Data Availability

The raw data from the dynamic trials reported in this study will be made available in an anonymized form as part of the European Musculoskeletal Database (EMD), established by the SmILE project. Access to the data will be facilitated through the project’s designated repository once data curation, quality checks, and anonymization processes are completed. In the interim, the full dataset can be made available to researchers upon reasonable request to the corresponding author, participant to the informed consent provided by the study participants.
